# Survival and complications of cytoreductive surgery with hyperthermic intraperitoneal chemotherapy in patients with intra-abdominal malignancies: A meta-analysis of randomized controlled trials

**DOI:** 10.3389/fphar.2023.1094834

**Published:** 2023-03-09

**Authors:** Changchun Jian, Hai Mou, Ye Zhang, Qingxin Fan, Yunsheng Ou

**Affiliations:** ^1^ Department of Orthopedics, The First Affiliated Hospital of Chongqing Medical University, Chongqing, China; ^2^ Orthopedic Laboratory of Chongqing Medical University, Chongqing, China

**Keywords:** cytoreductive surgery, hyperthermic intraperitoneal chemotherapy, intra-abdominal malignancies, peritoneal metastases, meta-analysis

## Abstract

**Background:** Peritoneal metastasis (PM) is an advanced stage of intra-abdominal malignancy with a very poor prognosis. In recent years, hyperthermic intraperitoneal chemotherapy (HIPEC) combined with cytoreductive surgery (CRS) has been utilized as an active treatment in the prevention and treatment of PM, with encouraging results. However, compared with CRS alone, the results of the CRS plus HIPEC strategy in the treatment of patients with intra-abdominal malignancies are still controversial. This study sought to determine the impact of HIPEC + CRS on patient survival and adverse events (AEs) by reviewing randomized controlled trials (RCTs) for all types of intra-abdominal malignancies.

**Methods:** A PubMed, Embase, Cochrane Library, Web of Science and Clinical Trials.gov search extracted all RCTs until 12 October 2022, examining the CRS + HIPEC vs. CRS alone strategies in the treatment of various types of intra-abdominal malignancies. The outcomes included overall survival (OS), disease-free survival (DFS), relapse-free survival (RFS), progression-free survival (PFS) and AEs. The dichotomous data were pooled and reported as odds ratios (ORs) with 95% confidence intervals (CIs). The survival outcome data were pooled using hazard ratios (HRs) and corresponding 95% CIs. The Cochrane Collaboration’s Risk of Bias Tool was used to assess the risk of bias in the included studies.

**Results:** A total of 12 RCTs were included in this meta-analysis, including 873 patients in the CRS + HIPEC group and 878 patients in the CRS alone group. The studies included 3 (617 patients) on colorectal cancer, 4 (416 patients) on gastric cancer, and 5 (718 patients) on ovarian cancer. Our analysis showed no difference in OS between the CRS + HIPEC and CRS alone groups (HR: 0.79, 95% CI 0.62–1.01). Subgroup analysis showed that CRS + HIPEC improved the OS of gastric cancer patients (HR: 0.49, 95% CI 0.32–0.76) compared with CRS alone. However, CRS + HIPEC did not significantly improve the OS of colorectal cancer (HR: 1.06, 95% CI 0.81–1.38) and ovarian cancer (HR: 0.82, 95% CI 0.62–1.07) patients. In addition, there was no significant difference in DFS/RFS (HR: 0.78, 95% CI 0.57–1.07) or PFS (HR: 1.03, 95% CI 0.77–1.38) between the two groups. Compared with CRS alone, CRS with HIPEC had greater nephrotoxicity (OR: 0.45, 95% CI 0.21–0.98), while other AEs did not differ significantly between the two groups.

**Conclusion:** Our results suggest that CRS + HIPEC may improve OS in gastric cancer patients compared with CRS alone, but we did not observe a benefit for DFS/RFS. For patients with ovarian and colorectal cancers, our results suggest that HIPEC + CRS does not appear to improve survival outcomes. In addition, CRS + HIPEC has higher nephrotoxicity than CRS alone. More evidence from RCTs is needed to evaluate whether the use of CRS + HIPEC is an appropriate option.

## Introduction

Advanced intra-abdominal malignancies are prone to peritoneal metastasis (PM), namely, peritoneal carcinomatosis, which is characterized by cancer metastasis to the peritoneal surface and the spread of malignant tumors in the peritoneal cavity (Coccolini et al., 2013). PM was previously considered to be a fatal disease with little opportunity for a cure because it has a lower response rate to normal systemic chemotherapy than other organ-specific metastases (McMullen et al., 2017).

However, over the past 3 decades, there has been a clear conceptual change in the prospects for PM prevention and treatment. Based on the concept that the “peritoneum is an organ”, PM is now widely considered to be a regional cancer metastasis. In some selected cases, scientific integration of existing technologies and active treatment can not only effectively control disease progression but in some patients may also achieve a clinical cure. Cytoreductive surgery (CRS) is considered the cornerstone of the treatment of patients with PM or for those at high risk of subsequent PM. The principle of CRS is to remove visible lesions, control macroscopic lesions, and achieve radical treatment at the histological level. CRS is considered a prerequisite for intraperitoneal chemotherapy (Glockzin and Piso, 2012).

Traditional intraperitoneal chemotherapy can effectively combat the microseeding and cancer cell embedding of intra-abdominal malignancies. In contrast, hyperthermic intraperitoneal chemotherapy (HIPEC) has a range of advantages. When administered after complete CRS, not only does the entire peritoneal surface become exposed to the chemotherapy drug but the effect of hyperthermia (41°C–43°C, 30–120 min) can also be used to increase the cytotoxicity of the chemotherapy drugs (Hettinga et al., 1997; Zivanovic et al., 2015). This combination of HIPEC is an effective treatment strategy because it can change the permeability of the cell membrane and enhance drug absorption (Bozzetti et al., 2008). HIPEC delivers a radical cytological treatment to the patient with PM by directly acting on the cancer cells in the peritoneal cavity and the metastatic nodules on the peritoneal surface and destroying these cells through the cytotoxicity of drugs (Kobayashi and Kodera, 2017).

The main principle behind combining CRS with HIPEC is that CRS removes all visible PM and then HIPEC treats any remaining microscopic lesions (Elias et al., 2014). In recent years, the CRS + HIPEC strategy has been considered a therapeutic approach that can improve the survival rate and disease burden of patients with most abdominal malignancies (Chia et al., 2016; Goodman et al., 2016; Brandl and Prabhu, 2021; Kitai, 2021; Badgwell, 2022). Whether the use of HIPEC as a combination therapy after CRS provides additional survival benefits without increased adverse events (AEs) in patients with intra-abdominal malignancies, with or without PM, remains controversial. Therefore, we conducted a meta-analysis of randomized clinical trials (RCTs) to review and evaluate the impact of the CRS + HIPEC treatment strategy on the incidence of AEs and the survival of patients with intra-abdominal malignancies compared to the use of CRS alone.

## Materials and methods

This meta-analysis was carried out in accordance with the Preferred Reporting Items for Systematic Reviews and Meta-analyses (PRISMA) statement (Page et al., 2021).

### Data sources and search strategy

PubMed, Embase, Cochrane Library, Web of Science and ClinicalTrials.gov were searched from inception to October 12, 2022. Taking PubMed as an example, the complete search strategy is shown in Additional file: Supplementary Table S1. We also manually searched references from selected relevant studies to find other studies that met the inclusion criteria. For studies repeatedly published due to an increase in the number of patients or follow-up time, we evaluated only the most recent version. Any article identified as likely to meet our inclusion criteria was evaluated in its entirety.

### Inclusion and exclusion criteria

Two investigators independently screened all studies by reviewing the titles and abstracts. Disagreements were resolved through discussion with a third investigator. We included all intra-abdominal tumor types. The literature was selected with the following inclusion criteria: (1) patients with intra-abdominal malignancies; (2) RCTs comparing the outcomes of CRS + HIPEC vs. CRS alone; (3) overall survival (OS), disease-free survival (DFS), relapse-free survival (RFS), progression-free survival (PFS), or AE data were available; and (4) only articles in English were included.

Studies that did not fulfill the above-mentioned criteria were excluded. Specifically, the exclusion criteria included (1) non-RCTs, case-control studies, abstracts, letters, comments, case reports or series, etc.; (2) primary interventions consisting only of CRS without HIPEC or HIPEC without CRS; (3) studies with less than 10 patients; (4) research involving non-human subjects; and (5) studies containing incomplete survival data or data that could not be used for statistical analysis.

### Data extraction and quality evaluation

The data extracted into a standard Excel form included the first author’s name, year of publication, recruitment period, study location, cancer type, number of patients, HIPEC regimens (including drugs, duration and temperature), median follow-up time, survival outcomes and AEs. For survival data, we extracted hazard ratios (HRs) with 95% confidence intervals (CIs) from the included studies. If the HR and 95% CI were not directly reported, we extracted data from Kaplan-Meier curves by Engauge Digitizeit 4.1, and we calculated the HR and 95% CI as described by Tierney (Tierney et al., 2007).

We used the Cochrane Collaboration’s tool for evaluating the risk of bias in the included RCTs (Higgins et al., 2011).

### Statistical analysis

Analyses were performed using Stata 14.0 (StataCorp, College Station, TX) and R 4.1.3 software (R Foundation for Statistical Computing, Beijing, China, “meta”, “robvis” and “ggplot2” packages). The dichotomous data results were pooled and reported as odds ratios (ORs) with 95% CIs. The survival outcome data were pooled using HRs and corresponding 95% CIs. We used the Cochrane Q test and I2 statistic to test the heterogeneity across studies: if *p* < 0.1 or I^2^ > 50%, the result was considered to indicate significant heterogeneity. When heterogeneity was identified, the DerSimonian-Laird method for fitting the random-effects model was used to calculate the pooled ORs with 95% CIs; otherwise, a fixed-effects model (Mantel-Haenszel’s method) was used to pool the estimates. A *p*-value less than 0.05 was considered statistically significant. A funnel plot was used to assess the publication bias of the included literature. Sensitivity analysis was performed to detect whether a single study had significantly affected the pooled results by excluding one study at a time. Subgroup analyses were also performed to explore the sources of heterogeneity.

## Results

### Characteristics of the included studies

**FIGURE 1:**
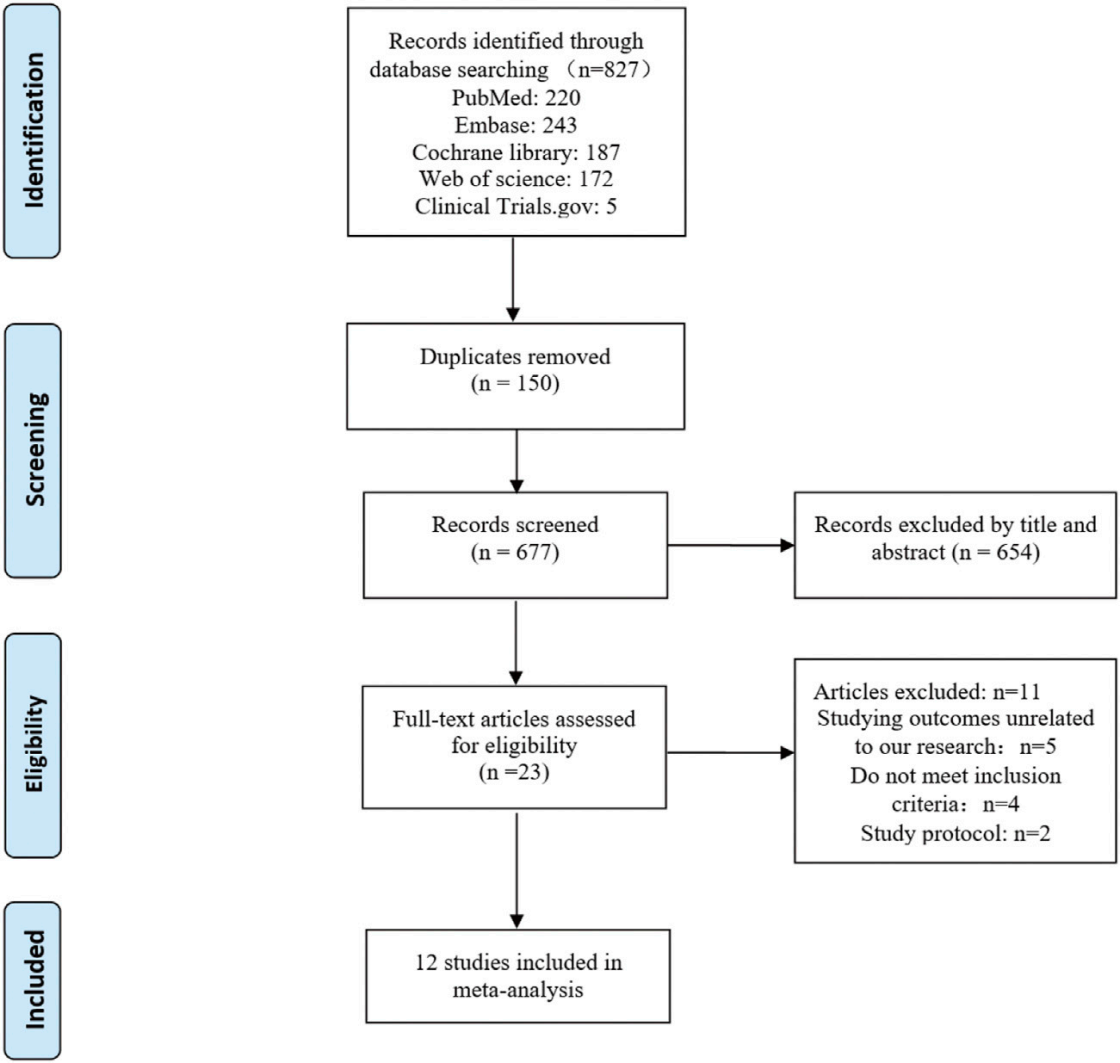
PRISMA flow chart of the screening strategy for the included studies.

Twelve studies published from 2011 to 2022 were included in the meta-analysis. These RCTs randomly assigned 1751 patients with intra-abdominal malignancies, including 873 in the CRS + HIPEC group and 878 in the CRS alone group. Cisplatin and oxaliplatin were the main chemotherapeutic agents used in the administration of HIPEC. The characteristics of the included studies are shown in Table 1. The study selection flowchart (PRISMA) is shown in Figure 1.

**TABLE 1:** Main characteristics of all RCTs included in the meta-analysis.

Author	Year	Recruitment period	Location	Cancer type	Group(n)		HIPEC regimens			MFU (m)
					CRS alone	CRS + HIPEC	Drugs	Duration (min)	Temp (°C)	
Klaver et al. (2019)	2019	2015–2017	Netherlands	Advanced primary CC without PM	102	100	Oxaliplatin (460 mg/m^2^)	30	42–43	23
Quénet et al. (2021)	2021	2008–2014	France	Advanced primary CC with synchronous PM	132	133	Oxaliplatin (360 mg/m^2^) closed or (460 mg/m^2^) open	30	43	63.8
Goéré et al. (2020)	2020	2010–2015	France	Advanced CC with synchronous PM or at high risk of subsequent PM	75	75	Oxaliplatin (460 mg/m^2^) or oxaliplatin (300 mg/m^2^) +irinotecan (200 mg/m^2^) or mitomycin (35 mg/m^2^)	30	42–44	50.8
Liu et al. (2022)	2022	2014–2018	China	GC stage-III	57	57	Cisplatin (30 mg/m^2^)	60	40–41	44
Yang et al. (2011)	2011	NA	China	GC with synchronous PM	34	34	Cisplatin 20 lg/mL, mitomycin C 5Lg/mL	60–90	42.5–43.5	32
Reutovich et al. (2019)	2019	2008–2016	Belarus	GC with synchronous PM	78	76	Cisplatin (50 mg/m^2^) + doxorubicin (50 mg/m^2^)	60	42	41
Beeharry et al. (2019)	2019	2014–2015	China	Advanced GC	40	40	Cisplatin (50 mg/m^2^)	60	41–43	32
van Driel et al. (2018)	2018	2007–2016	Netherlands	OC with PM	123	122	Cisplatin (100 mg/m^2^)	120	40	56.4
Antonio et al. (2022)	2022	2012–2018	Spain	OC with or without PM	36	35	Cisplatin (75 mg/m^2^)	60	42–43	32
Zivanovic et al. (2021)	2021	2014–2019	America	Recurrence of OC	49	49	Carboplatin (800 mg/m^2^)	90	41–43	39.5
Spiliotis et al. (2015)	2015	2006–2013	Greece	Recurrence of advanced OC	60	60	Cisplatin (100 mg/m^2^) and paclitaxel (175 mg/m^2^)	60	42.5	NA
Lim M. C. et al. (2022)	2022	2010–2016	South Korea	Advanced OC with or without PM	92	92	Cisplatin (75 mg/m^2^)	90	41.5	69.4

CC: colorectal cancer; GC: gastric cancer; OC: ovarian cancer; PM: peritoneal metastasis; CRS: cytoreductive surgery; HIPEC: hyperthermic intraoperative peritoneal chemotherapy; n: participants; NA: not available; MFU: median follow-up; m: month.

### OS

**FIGURE 2:**
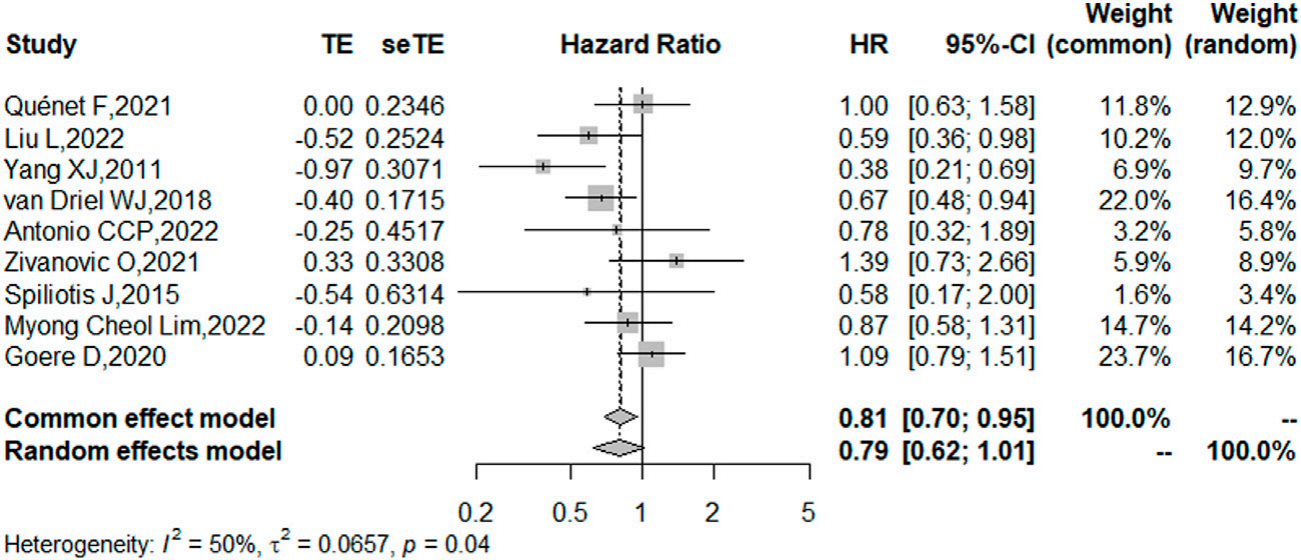
Forest plot of OS for patients with intra-abdominal malignancies treated with CRS + HIPEC vs. CRS alone.

**FIGURE 3:**
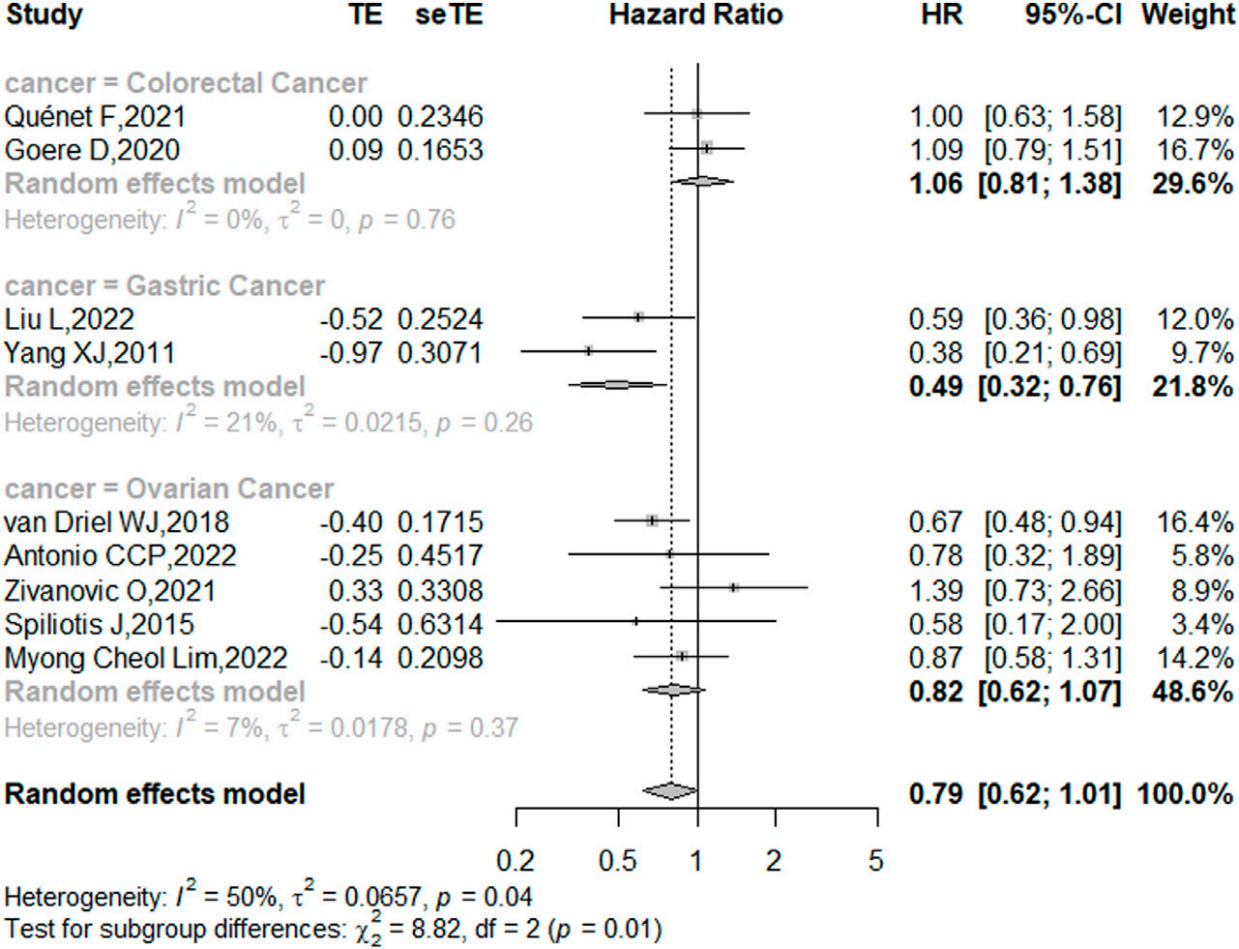
Subgroup analysis of OS based on patients with different types of intra-abdominal malignancies treated with CRS + HIPEC vs. CRS alone.

**FIGURE 4:**
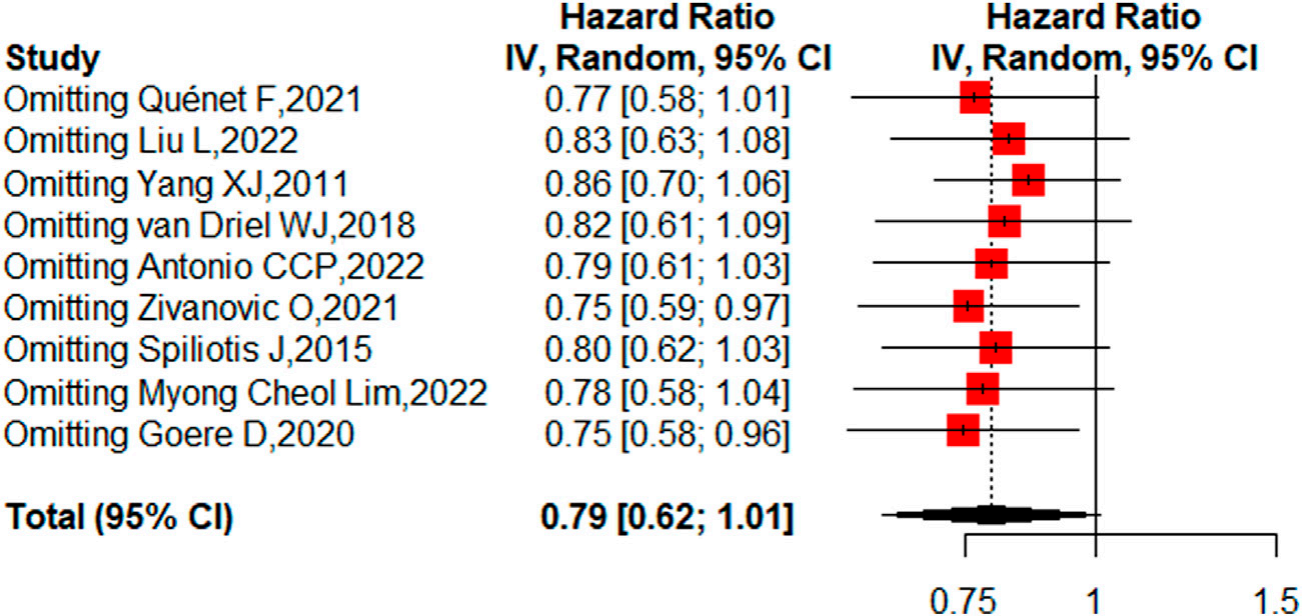
Sensitivity analysis of OS.

Nine studies reported the 5-year OS with or without HIPEC. This meta-analysis showed no difference in OS between the CRS + HIPEC and CRS alone groups (HRs: 0.79, 95% CI 0.62–1.01, Figure 2). Moderate heterogeneity was present across the studies (I^2^ = 50%; *p* = 0.04, Figure 2). To explore the source of the heterogeneity, we performed a subgroup analysis based on different cancer types (Figure 3). Subgroup analysis showed that the heterogeneity of colorectal cancer (Goéré et al., 2020; Quénet et al., 2021), gastric cancer (Yang et al., 2011; Liu et al., 2022) and ovarian cancer (Spiliotis et al., 2015; van Driel et al., 2018; Zivanovic et al., 2021; Antonio et al., 2022; Lim M. C. et al., 2022) was low (I^2^ = 0%, 21% and 7%, respectively). In addition, the subgroup analysis showed that the HRs (95% CIs) of colorectal cancer and ovarian cancer were 1.06 (0.81–1.38) and 0.82 (0.62–1.07), respectively. The results suggested that HIPEC did not seem to significantly improve the OS of colorectal cancer and ovarian cancer patients. However, the results suggest that CRS + HIPEC may improve the OS of gastric cancer (HR: 0.49, 95% CI 0.32–0.76) compared with CRS alone. Although we included only 2 RCT studies on gastric cancer in the analysis, this result was consistent with the findings of Bonnot et al. (Bonnot et al., 2019). We also performed sensitivity analysis to explore the source of the heterogeneity. The results of the sensitivity analysis are presented in Figure 4.

### DFS/RFS

**FIGURE 5:**
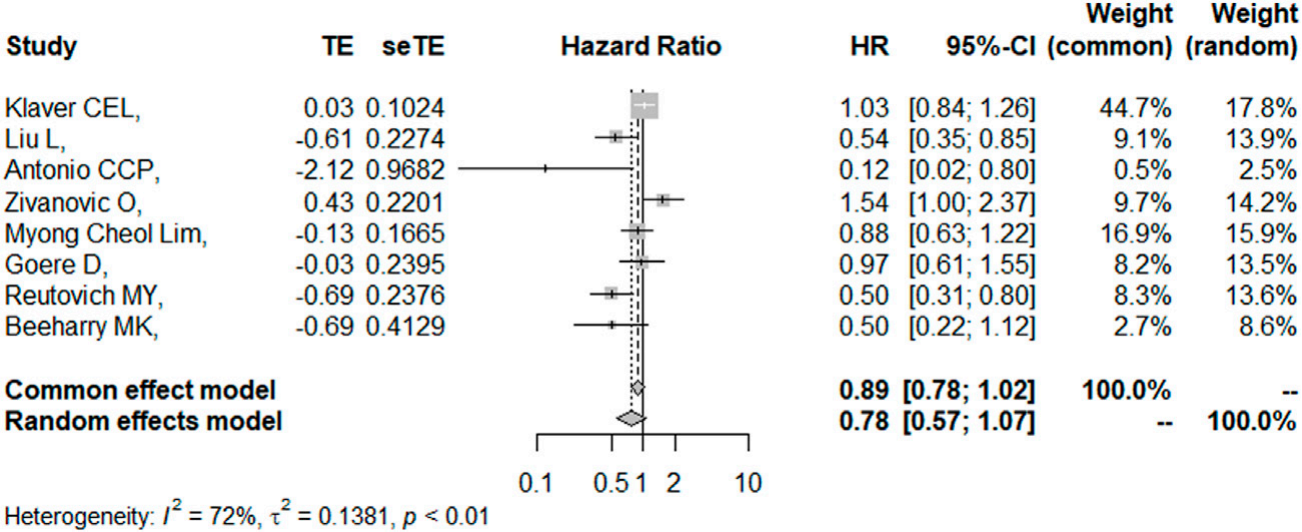
Forest plot of DFS/RFS.

**FIGURE 6:**
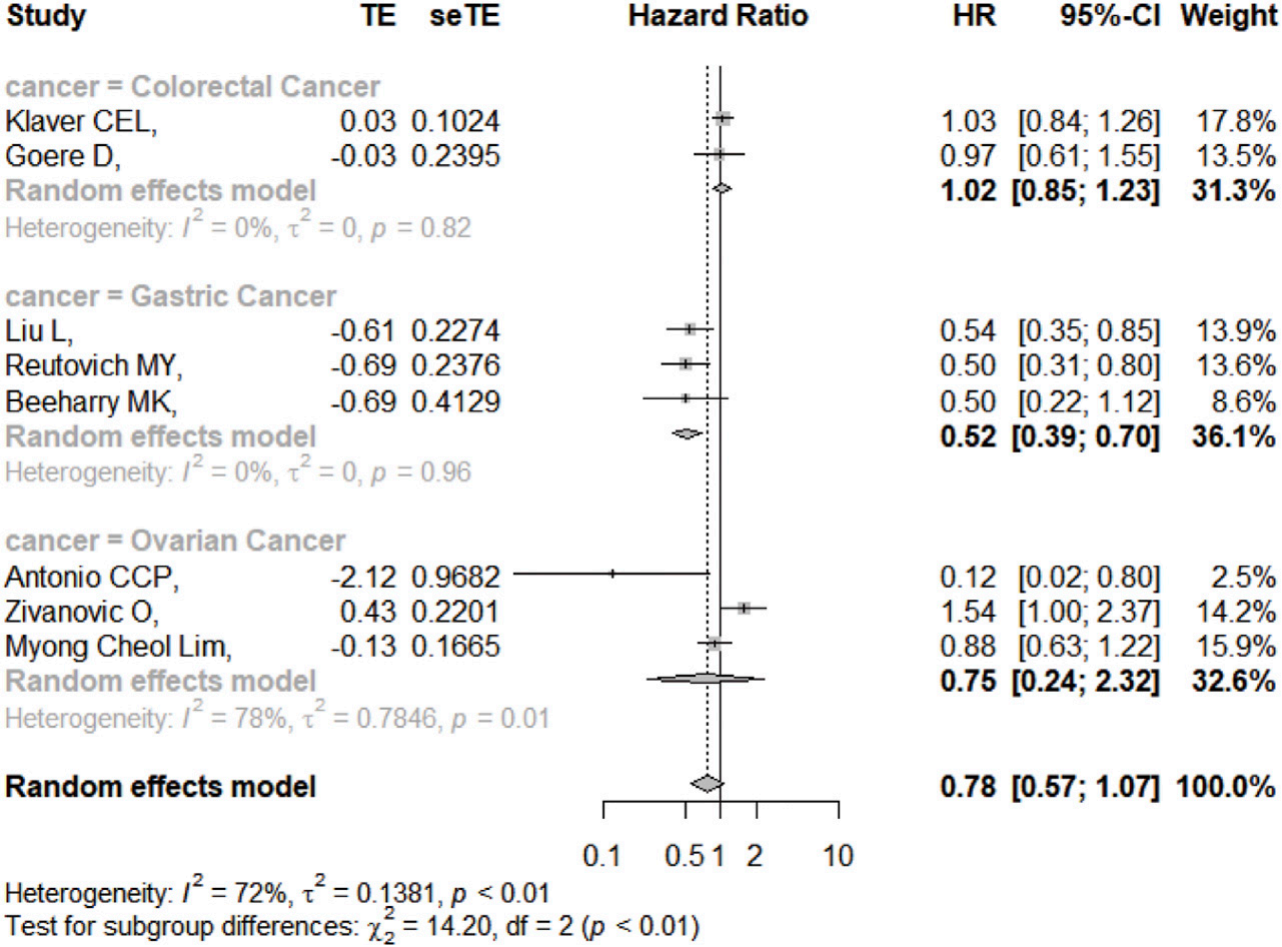
Subgroup analysis of DFS/RFS based on different cancers.

**FIGURE 7:**
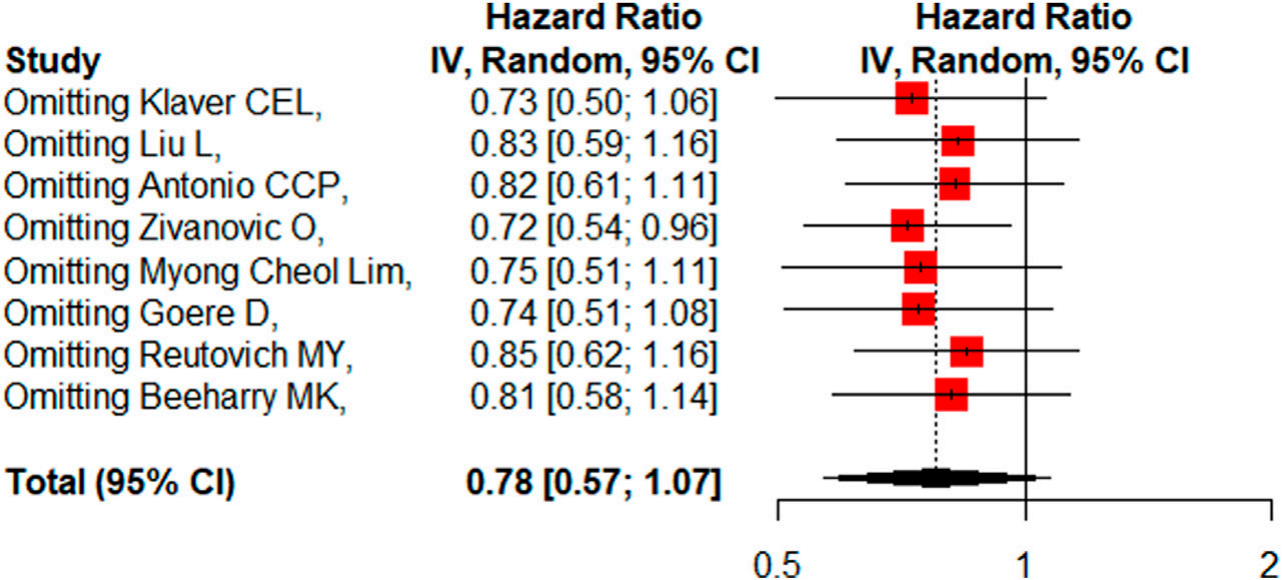
Sensitivity analysis of DFS/RFS.

Eight studies reported the 5-year DFS/RFS with or without HIPEC. This meta-analysis showed no significant difference between the CRS + HIPEC and CRS alone groups (HR: 0.78, 95% CI 0.57–1.07, Figure 5). High heterogeneity was present across the studies (I^2^ = 72%; *p* < 0.01, Figure 5). To explore the source of heterogeneity, we performed a subgroup analysis based on different cancers (Figure 6). Subgroup analysis showed that there was no heterogeneity between colorectal cancer (Klaver et al., 2019; Goéré et al., 2020) and gastric cancer studies (Beeharry et al., 2019; Reutovich et al., 2019; Liu et al., 2022) (I^2^ = 0%). There was high heterogeneity (I^2^ = 78%) among the three studies on ovarian cancer (Zivanovic et al., 2021; Antonio et al., 2022; Lim M. C. et al., 2022). We also performed sensitivity analysis to explore the source of the heterogeneity. The results of the sensitivity analysis are presented in Figure 7. Based on the results of the sensitivity analysis and subgroup analysis, the study of Zivanovic O (Zivanovic et al., 2021)was determined to have great heterogeneity.

### PFS

**FIGURE 8:**
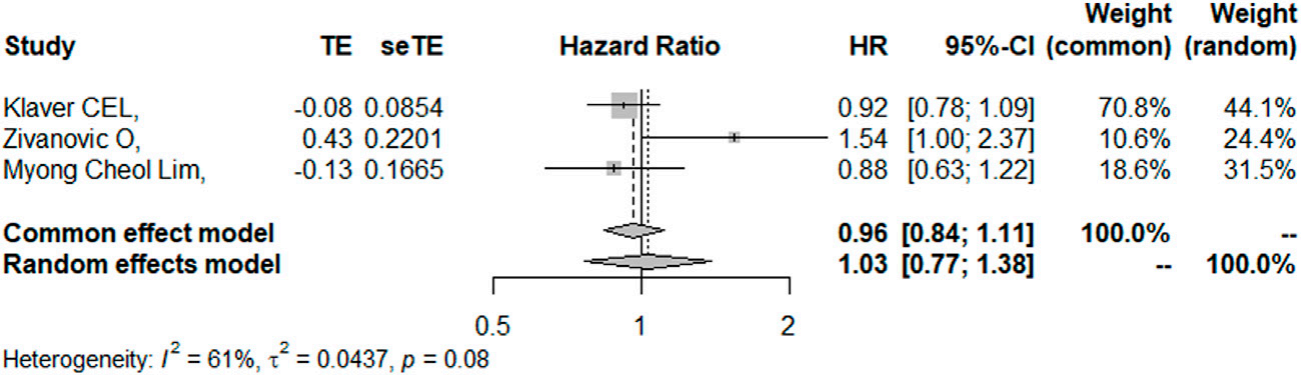
Forest plot of PFS.

**FIGURE 9:**
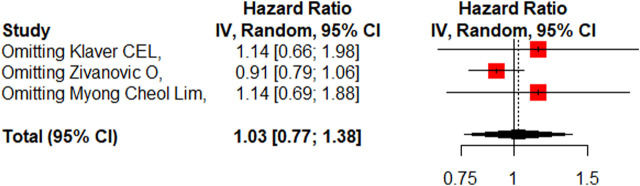
Sensitivity analysis of PFS.

Three studies (Klaver et al., 2019; Zivanovic et al., 2021; Lim M. C. et al., 2022) reported the 5-year PFS with or without HIPEC. This meta-analysis showed no significant difference between the CRS + HIPEC and CRS alone groups (HR: 1.03, 95% CI 0.77–1.38, Figure 8). High heterogeneity was present across the studies (I^2^ = 61%; *p* = 0.08, Figure 8). To explore the source of heterogeneity, we performed a sensitivity analysis. The results of the sensitivity analysis are presented in Figure 9. As there were only three articles included in this part of the analysis, the source of heterogeneity could not be completely explored, so we adopted the random effect model for statistical analysis.

### AEs

**FIGURE 10:**
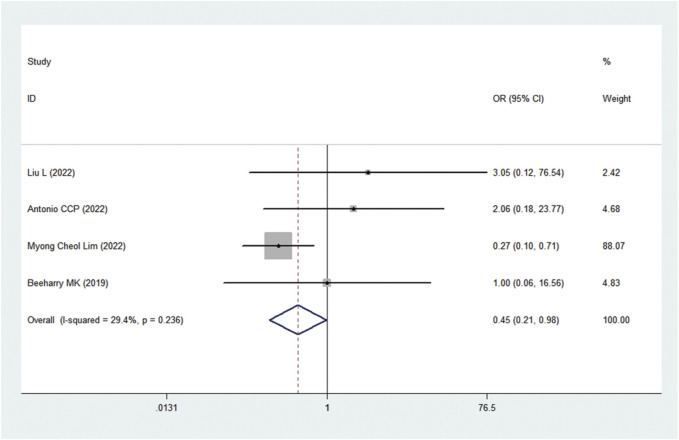
Forest plot of AEs: renal dysfunction.

**FIGURE 11:**
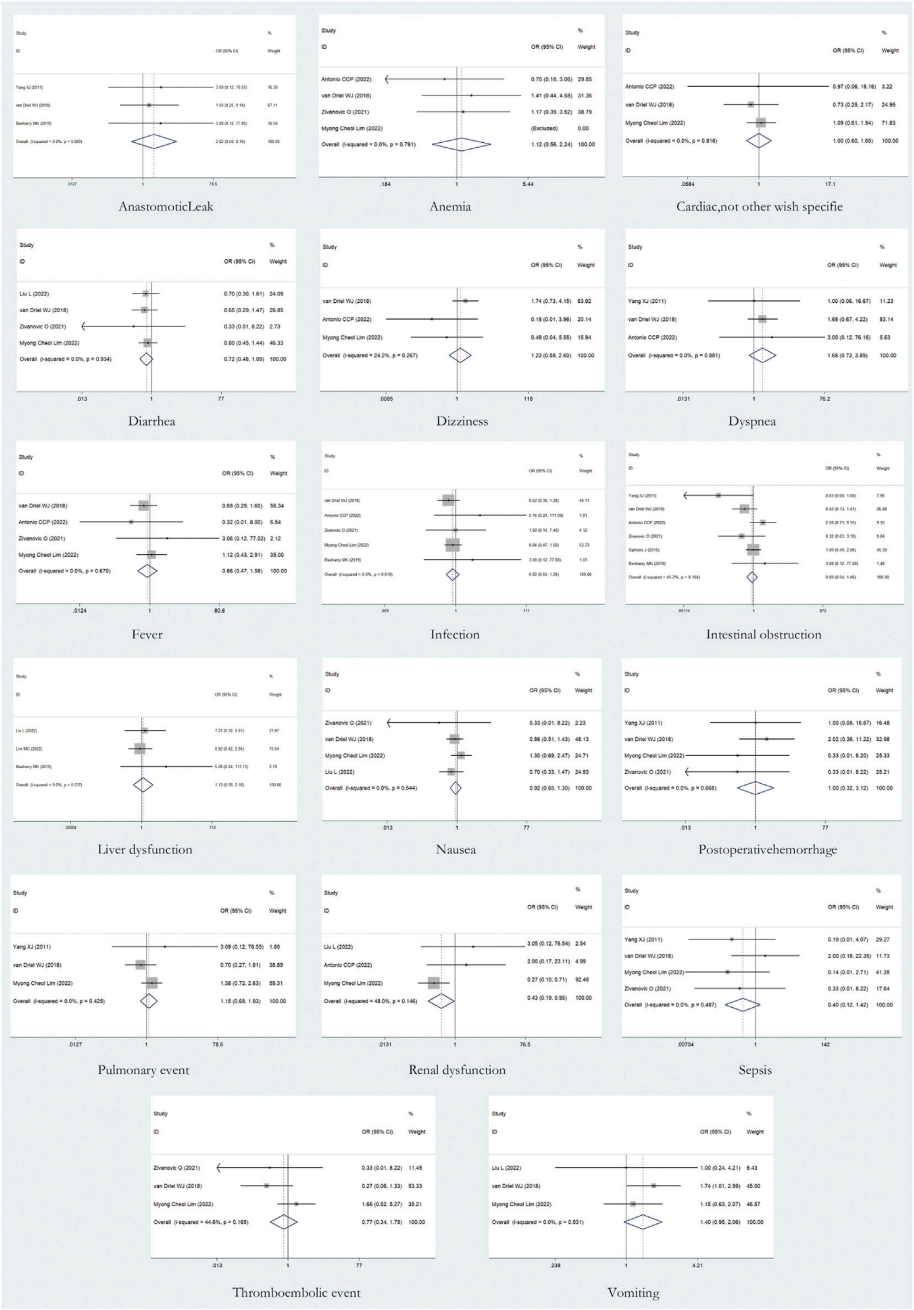
Forest plot of AEs: other AEs.

A total of 17 AEs were available for statistical analysis in the 12 included RCTs. Our statistical analysis results showed that compared with CRS alone, CRS + HIPEC has greater nephrotoxicity (i.e., renal insufficiency) (OR: 0.45, 95% CI 0.21–0.98, Figure 10). Mild heterogeneity was present across the studies (I^2^ = 29.4%; *p* = 0.24, Figure 10). However, there were no significant differences in other AEs between the two groups, as shown in Figure 11. Mild or no heterogeneity was present, as shown in Figure 11. Nine studies (Yang et al., 2011; van Driel et al., 2018; Beeharry et al., 2019; Klaver et al., 2019; Reutovich et al., 2019; Quénet et al., 2021; Zivanovic et al., 2021; Antonio et al., 2022; Lim M. C. et al., 2022) with a total of 1,362 patients (685 in the CRS group and 677 in the CRS + HIPEC group) provided data on grade ≥3 AEs. The percentage of patients who had grade ≥3 AEs was similar between the two groups, as shown in additional file: Supplementary Table S2.

### Publication bias

**FIGURE 12:**
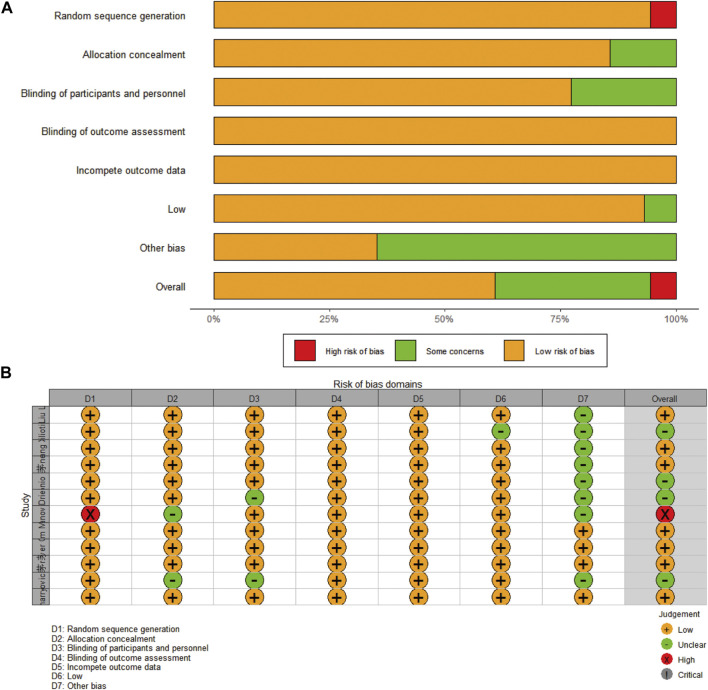
Risk of bias of the included studies: **(A)** presented as percentages across all included studies; **(B)** based on the Cochrane Collaboration’s Risk of Bias Tool.

Funnel plots were used to assess the publication bias of the included literature, and funnel plots of survival outcomes and AEs are shown in additional file: Supplementary Figures S1, S2, respectively. The Cochrane Collaboration’s Risk of Bias Tool was used for RCTs. The results are shown in Figure 12.

## Discussion

In the past two to 3 decades, trials on CRS + HIPEC have reported varying degrees of improvement in patient survival, tumor recurrence, and PM. For example, HIPEC significantly improved OS and/or PFS in patients with primary ovarian cancer compared with patients who did not receive HIPEC (Wu et al., 2019; Cianci et al., 2020). Despite these findings, the medical community is still not fully convinced of the efficacy and safety of CRS + HIPEC because most of the data are from cohort studies. This implies the need for evidence based on RCTs. Therefore, our meta-analysis included only published RCTs on CRS + HIPEC vs. CRS alone as treatment strategies in patients with intra-abdominal malignancies. Our analysis assessed the survival outcomes of these patients in terms of OS, DFS/RFS, or PFS, as well as AEs.

Various intra-abdominal malignancies are very difficult to treat once PM occurs. For a long period of time, only systemic chemotherapy and other palliative treatments have been used to prolong the patient’s life and improve the quality of life. However, for some patients, it is difficult for conventional hydrophilic drugs to reach the tumor tissue, possibly due to the existence of the “blood-peritoneal barrier”. Simply increasing the dose of chemotherapy drugs poses a serious health threat to these patients, and ultimately such patients do not respond well to systemic chemotherapy. Studies have shown that CRS can re-enter the proliferative phase of the cell cycle, thereby increasing cell sensitivity to anticancer drugs (Yan et al., 2010). HIPEC prevents and treats PM by combining three main mechanisms: chemotherapy, hyperthermia and mechanical flushing (Sugarbaker, 2005; Garofalo et al., 2006; Chiva and Gonzalez-Martin, 2015). Because the tolerance of normal cells and tumor cells to temperature is different (Garofalo et al., 2006), thermochemotherapeutic drugs can directly contact tumor cells in the peritoneum and produce cytotoxicity after convection and diffusion (Lim P. Q. et al., 2022). HIPEC can eradicate single cancer cells or microscopic nodules, but because the peritoneal penetration of chemotherapy drugs is limited, the tumor must be eliminated as thoroughly as possible first. With CRS alone or HIPEC alone, it is difficult to prevent or treat PM in gastrointestinal or ovarian cancer. In recent years, CRS + HIPEC has generally been considered an aggressive palliative care option in the hope of providing more survival benefits to patients with intra-abdominal malignancies.

Many planned or ongoing RCTs have attempted to analyze the efficacy and safety of CRS + HIPEC vs. CRS alone for the treatment or prevention of PM. We aimed to determine whether CRS + HIPEC is applicable to all types of tumors in the abdominal cavity, but to date, we retrieved only 12 published RCTs, including three on colorectal cancer, four on gastric cancer, and five on ovarian cancer. No RCTs were available for pancreatic cancer and other primary abdominal malignancies, such as pseudomyxoma peritoneum and malignant peritoneal mesothelioma.

Previous meta-analyses of CRS + HIPEC for the prevention and treatment of PM have mainly involved cohort studies, and the use of this strategy in patients with intra-abdominal tumors seems promising. However, our RCT-based analysis showed that the introduction of HIPEC after CRS did not provide additional DFS/RFS or PFS benefits in patients with intra-abdominal malignancies. Moderate heterogeneity of OS was observed. Sensitivity analysis did not show that any study significantly affected the statistical results. Subgroup analysis based on the cancer type suggested that CRS + HIPEC did not improve OS in patients with colorectal cancer and ovarian cancer compared with CRS alone but seemed to improve OS in patients with gastric cancer. Consistent with our findings, a meta-analysis of gastrointestinal tumors suggested that in patients with gastric cancer, peritoneal recurrence-free survival was significantly higher in the group that received HIPEC + CRS; however, there was no significant improvement in peritoneal-free survival in patients with colorectal cancer (Dominic et al., 2021). For DFS/RFS, the reason for the greater heterogeneity may be that Zivanovic O (Zivanovic et al., 2021) included patients with recurrent ovarian cancer, while the other two studies included patients with primary advanced ovarian cancer. A previous meta-analysis suggested that CRS + HIPEC could significantly improve OS and DFS in ovarian cancer patients treated with neoadjuvant chemotherapy but had no significant benefits for the prognosis of primary ovarian cancer patients who did not receive neoadjuvant chemotherapy or patients with recurrent ovarian cancer (Filis et al., 2022). This is consistent with the study of Lim et al. (Lim M. C. et al., 2022). Whether there is a difference in the effectiveness of HIPEC for recurrent and primary ovarian cancer is still inconclusive and more evidence is needed.

There are many factors influencing the prognosis of patients with intra-abdominal malignancies treated by HIPEC, such as histopathological type, stage and drugs used in HIPEC. In the literature included in our meta-analysis, advanced epithelial ovarian cancer (EOC) and recurrent ovarian cancer were included. For patients with gastric and colorectal cancer, the included RCTs were not stratified according to histopathological type and stage. The study by Bae JH et al. (Bae et al., 2007) used carboplatin or paclitaxel in HIPEC for patients with EOC and compared them with the control group; both the carboplatin-HIPEC and paclitaxel-HIPEC groups had a better prognosis than the control group, but there was no significant difference in efficacy between paclitaxel and carboplatin. Unfortunately, in the 12 RCTs included in this study, there were no available data to analyze the effects of different drugs used in HIPEC on patient prognosis. At present, there are few high-quality RCTs on the effects of different histopathological types, stages and HIPEC drugs on the prognosis of gastric cancer, colorectal cancer, and ovarian cancer. Thus, more evidence is needed.

AEs such as respiratory, liver, or renal failure and infection after HIPEC are troubling problems for surgeons, and the potential risk of death and long-term sequelae of the procedure masks the potential use of HIPEC. The drugs for HIPEC are mostly first-line platinum drugs. In this meta-analysis, we found that the drug for HIPEC used in colorectal cancer was oxaliplatin, and the main drug for HIPEC used in gastric cancer and ovarian cancer was cisplatin (as shown in Table 1). Our statistical results indicate that CRS + HIPEC appears to have higher nephrotoxicity than CRS alone in cases of abdominal malignancy. This may be because cisplatin is used in most HIPEC strategies, and intraperitoneal cisplatin is absorbed and circulated throughout the body, resulting in nephrotoxicity. Recent data show that approximately 3.7% of patients receiving HIPEC plus cisplatin have acute renal injury (Hakeam et al., 2014). However, the risk of other AEs was comparable between the CRS + HIPEC and CRS alone groups, and the percentage of patients who had grade ≥3 AEs was similar between the two groups (as shown in additional file: Supplementary Table S2). A controlled study by Ghirardi V et al. also suggested that HIPEC is feasible in ovarian cancer patients and does not increase perioperative complications (Ghirardi et al., 2020). This may be because the majority of such RCTs are performed in clinical centers with extensive experience with HIPEC, and the incidence of postoperative complications may be related to the experience of institutions newly implementing the technique (Bakrin et al., 2013; Moran et al., 2015; Passot et al., 2016). Therefore, HIPEC should be considered a safe treatment modality for both clinical practice and research applications in experienced institutions.

Our study also had some limitations. A limited number of RCTs were included, and relatively few survival and AE data were available for analysis. Second, our decision to select high-quality evidence meant that we included only RCTs in the meta-analysis. Although RCTs are considered to be the highest level of clinical evidence available, they may interfere with the results because RCT study selection or inclusion criteria may be biased towards patients with a better disease biology or burden. In addition, too many bias factors (different drugs and dosages, different infusion times at different temperatures, different administration times, different histopathological types and different disease stages, etc.) can affect the conclusion of HIPEC treatment for pelvic and abdominal malignancies, so more high-quality studies are needed in the future to provide evidence for clinical practice.

## Conclusion

Considering the current RCT evidence, CRS + HIPEC may improve OS in gastric cancer patients compared with CRS alone, but we did not observe a benefit for DFS/RFS. For patients with ovarian and colorectal cancer, our results suggest that HIPEC + CRS does not appear to improve survival outcomes. In addition, CRS + HIPEC has higher nephrotoxicity than CRS alone. More evidence from RCTs is needed to evaluate whether the use of CRS + HIPEC is an appropriate option.

## Data Availability

The original contributions presented in the study are included in the article/Supplementary Material, further inquiries can be directed to the corresponding author.

## Supplementary material

Supplementary Figure S1 Funnel plots of survival outcomes: **(A)** OS; **(B)** DFS/RFS; **(C)** PFS.

Supplementary Figure S2 Funnel plots of AEs.
