# Supplementary material for: Survival and complications of cytoreductive surgery with hyperthermic intraperitoneal chemotherapy in patients with intra-abdominal malignancies: A meta-analysis of randomized controlled trials
Source: Front Pharmacol. 2023 Mar 9;14:1094834. doi: 10.3389/fphar.2023.1094834 (PMC10036049; doi:10.3389/fphar.2023.1094834)
Supplement: Supplementary file 1 [file Table1.docx]

**Figure and table legends:**

**Figure S1:** Funnel plots of survival outcomes: (A) OS; (B) DFS/RFS; (C) PFS.

**Figure S2:** Funnel plots of AEs.

**Table S1:** PubMed search strategy.

**Table S2:** Percentage of patients who had grade ≥3 AEs in the CRS and CRS + HIPEC groups.

**Table S1:** PubMed search strategy

| #1 | "Neoplasms"[Mesh] |
| --- | --- |
| #2 | (((((Tumor*[Title/Abstract]) OR (Neoplas*[Title/Abstract])) OR (Cancer*[Title/Abstract])) OR (Malignant Neoplasm*[Title/Abstract])) OR (Malignanc*[Title/Abstract])) OR (Benign Neoplasm*[Title/Abstract]) |
| #3 | "Cytoreduction Surgical Procedures"[Mesh] |
| #4 | ((Cytoreducti* Surgical Procedure*[Title/Abstract]) OR (Debulking Surgical Procedure*[Title/Abstract])) OR (Cytoreductive Surger*[Title/Abstract]) |
| #5 | "Hyperthermic Intraperitoneal Chemotherapy"[Mesh] |
| #6 | (((Hyperthermic Intraperitoneal Chemotherapy [Title/Abstract]) OR (HIPEC[Title/Abstract])) OR (Hot Chemotherapy [Title/Abstract])) OR (Intraperitoneal Hyperthermic Chemotherap*[Title/Abstract]) |
| #7 | randomized controlled trial [Publication Type] OR randomized [Title/Abstract] |
| #8 | #1 OR #2 |
| #9 | #3 OR #4 |
| #10 | #5 OR #6 |
| #11 | #8 AND #9 AND #10 AND #7 |
