# Supplementary figures and images for: Survival and complications of cytoreductive surgery with hyperthermic intraperitoneal chemotherapy in patients with intra-abdominal malignancies: A meta-analysis of randomized controlled trials
Source: Front Pharmacol. 2023 Mar 9;14:1094834. doi: 10.3389/fphar.2023.1094834 (PMC10036049; doi:10.3389/fphar.2023.1094834)

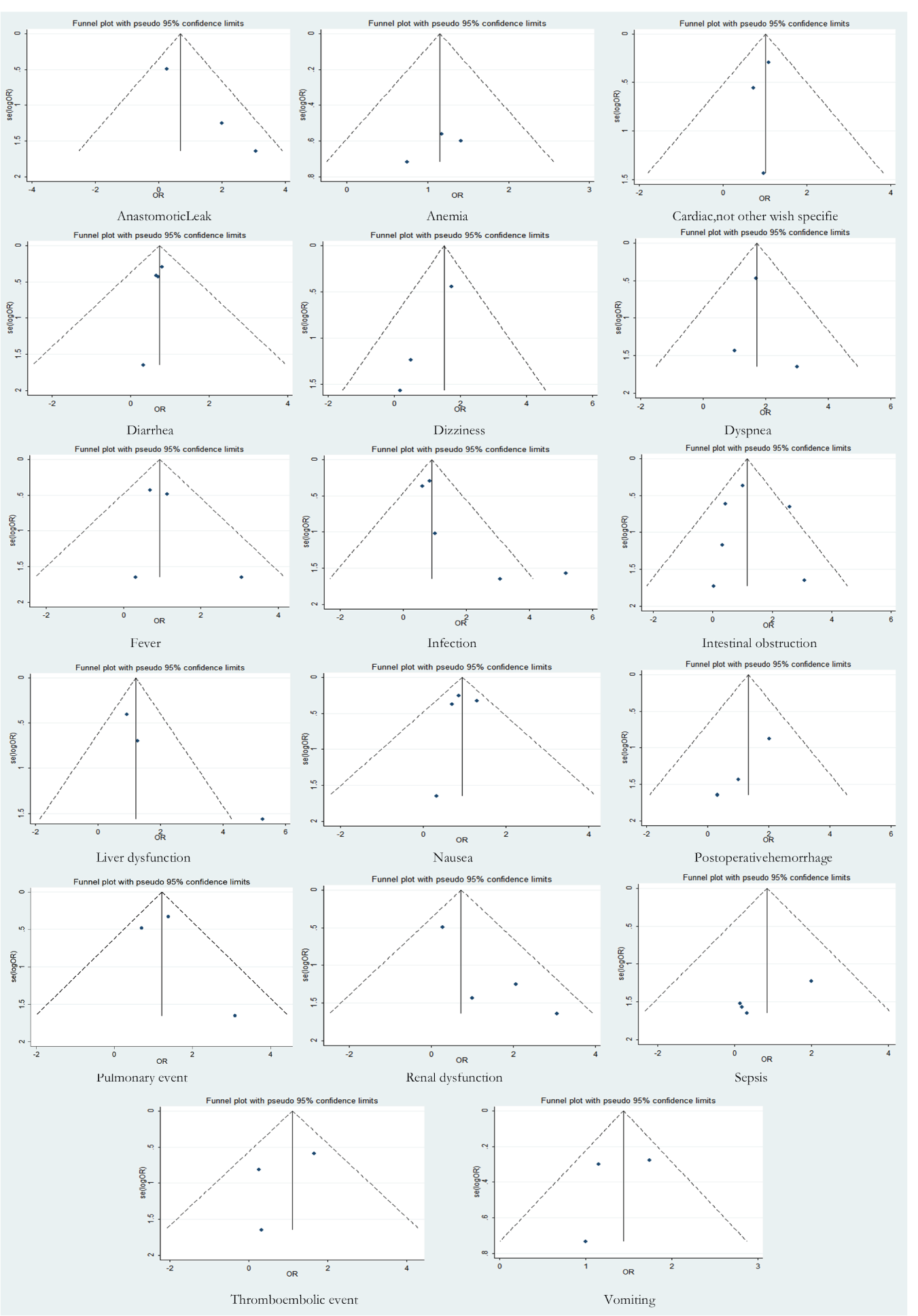

Supplement: Supplementary file 2 [file Image2.TIF]

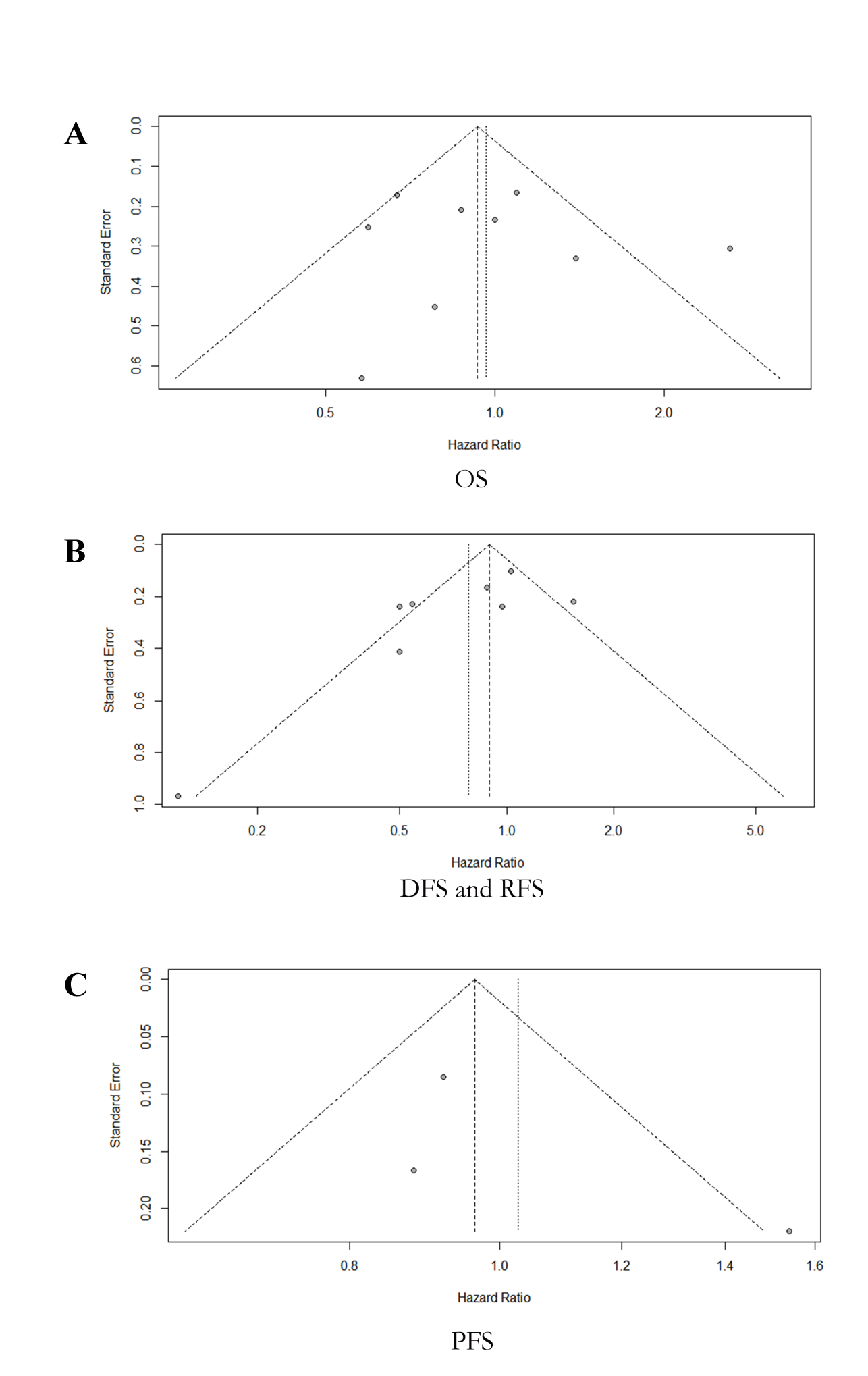

Supplement: Supplementary file 3 [file Image1.TIF]
