# Supplementary material for: Survival and complications of cytoreductive surgery with hyperthermic intraperitoneal chemotherapy in patients with intra-abdominal malignancies: A meta-analysis of randomized controlled trials
Source: Front Pharmacol. 2023 Mar 9;14:1094834. doi: 10.3389/fphar.2023.1094834 (PMC10036049; doi:10.3389/fphar.2023.1094834)
Supplement: Supplementary file 4 [file Table2.docx]

**Table S2:** Percentage of patients who had grade ≥3 AEs in the CRS and CRS + HIPEC groups.

| **Author** | **Year of publication** | **CRS alone group(N)** | **CRS+HIPEC group(N)** | **Graded≥3** | | **P value** |
| --- | --- | --- | --- | --- | --- | --- |
|  |  |  |  | **CRS** | **CRS+HIPEC** |  |
|  |  |  |  | **n /N (%)** | **n /N (%)** |  |
| Klaver et al. (2019) | 2019 | 102 | 100 | 3 /102=3% | 10/100=10% | NA |
| Quénet et al. (2021) | 2021 | 132 | 133 | 42/132=32% | 56/133=42% | p = 0·083 |
| Yang et al. (2011) | 2011 | 34 | 34 | 4/34=11.7% | 5/34=14.7% | P = 0.839 |
| Reutovich et al. (2019) | 2019 | 78 | 76 | 11/78=14.1% | 13/76=17.1% | P = 0.254 |
| Beeharry et al. (2019) | 2019 | 40 | 40 | 11/40=27.5% | 3/40=7.5% | P = 0.556 |
| van Driel et al. (2018) | 2018 | 123 | 122 | 31/122=25% | 32/118=27% | P = 0.76***** |
| Antonio et al. (2022) | 2022 | 36 | 35 | 10/36=27.8% | 10/35=28.6% | P＞0.05 |
| Zivanovic et al. (2021) | 2021 | 49 | 49 | 10/49=20% | 12/49=24% | P = 0.81 |
| Lim M. C. et al. (2022) | 2022 | 92 | 92 | 80/92=87.0% | 86/92=93.5% | NA |
|  |  |  | total: | 202/685=29.5% | 227/677=33.5% | 0.88 |

Note: CRS: cytoreductive surgery; HIPEC: hyperthermic intraoperative peritoneal chemotherapy; N: participants; n: occurrences; NA: not available; ***:** Five patients (one in the CRS group and four in the CRS+HIPEC group) were excluded from the safety analysis owing to not receiving assigned treatment.
